# Biochanin A Improves Memory Decline and Brain Pathology in Cuprizone-Induced Mouse Model of Multiple Sclerosis

**DOI:** 10.3390/bs12030070

**Published:** 2022-03-04

**Authors:** Rahaf Saeed Aldhahri, Badrah Saeed Alghamdi, Noor Ahmed Alzahrani, Khulud Abdullah Bahaidrah, Hadeil Muhanna Alsufiani, Rasha Abdulrashed Mansouri, Ghulam Md Ashraf

**Affiliations:** 1 Department of Biochemistry, Faculty of Sciences, King Abdulaziz University, Jeddah 21589, Saudi Arabia; raldhahri0020@stu.kau.edu.sa (R.S.A.); nsalahalzahrani@stu.kau.edu.sa (N.A.A.); kahmedbahaidrah@stu.kau.edu.sa (K.A.B.); halsufiani@kau.edu.sa (H.M.A.); 2 Department of Biochemistry, Faculty of Sciences, University of Jeddah, Jeddah 21959, Saudi Arabia; 3 Pre-Clinical Research Unit, King Fahd Medical Research Center, King Abdulaziz University, Jeddah 21589, Saudi Arabia; basalghamdi@kau.edu.sa; 4 Neuroscience Unit, Department of Physiology, Faculty of Medicine, King Abdulaziz University, Jeddah 21589, Saudi Arabia; 5 Department of Medical Laboratory Technology, Faculty of Applied Medical Sciences, King Abdulaziz University, Jeddah 21589, Saudi Arabia

**Keywords:** biochanin A, cognitive impairment, cuprizone, memory decline, multiple sclerosis

## Abstract

Multiple sclerosis (MS) is a chronic inflammatory and neurodegenerative disease of the central nervous system characterized by the demyelination of nerves, neural degeneration, and axonal loss. Cognitive impairment, including memory decline, is a significant feature in MS affecting up to 70% of patients. Thereby, it substantially impacts patients’ quality of life. Biochanin A (BCA) is an o-methylated isoflavone with a wide variety of pharmacological activities, including antioxidant, anti-inflammatory, and neuroprotective activities. Thus, this study aimed to investigate the possible protective effects of BCA on memory decline in the cuprizone (CPZ) model of MS. Thirty Swiss albino male mice (SWR/J) were randomly divided into three groups (n = 10): control (normal chow + i.p. 1:9 mixture of DMSO and PBS), CPZ (0.2% *w*/*w* of CPZ mixed into chow + i.p. 1:9 mixture of DMSO and PBS), and CPZ + BCA (0.2% *w*/*w* of CPZ mixed into chow + i.p. 40 mg/kg of BCA). At the last week of the study (week 5), a series of behavioral tasks were performed. A grip strength test was performed to assess muscle weakness while Y-maze, novel object recognition task (NORT), and novel arm discrimination task (NADT) were performed to assess memory. Additionally, histological examination of the hippocampus and the prefrontal cortex (PFC) were conducted. BCA administration caused a significant increase in the grip strength compared with the CPZ group. Additionally, BCA significantly improved the mice’s spatial memory in the Y-maze and recognition memory in the NORT and the NADT compared with the CPZ group. Moreover, BCA mitigated neuronal damage in the PFC and the hippocampus after five weeks of administration. In conclusion, our data demonstrates the possible protective effect of BCA against memory deterioration in mice fed with CPZ for five weeks.

## 1. Introduction

Multiple sclerosis (MS) is a chronic inflammatory and neurodegenerative disease of the central nervous system (CNS) that is characterized by the demyelination of nerves, neural degeneration, and axonal loss [1]. The most common features of MS are motor, sensory, and cognitive deficits [2]. In fact, cognitive impairment is a significant feature in MS, affecting between 43–70% of patients [3,4]. It can occur at any time during the disease progression and substantially impact patients’ quality of life [5]. The most affected cognitive domains in MS are information processing speed (20–50%) and working memory (33–65%) [4]. Although the exact pathogenesis of cognitive impairment in MS is not fully understood, advanced MRI techniques attributed it to several elements, including white matter lesions, grey matter atrophy, and altered connectivity of grey matter structures such as the hippocampus and cerebral cortex [6]. In fact, the hippocampus and the prefrontal cortex (PFC) are the key memory processing brain regions and the proper communication between these two regions is significant for memory, learning, and cognition [7]. On the other hand, any disrupted communication between them is associated with cognitive deficits in several neurological conditions including MS [7,8]. Moreover, neuroinflammation and its related microglial-mediated response have been hypothesized to play a role in cognitive dysfunction in MS [9,10].

Cuprizone (CPZ) is a copper chelator used commonly to induce consistent demyelination in rodents [11]. Intoxication with CPZ disturbs energy metabolism in oligodendrocytes leading to their apoptosis and induces neuroinflammation in the brain by activating microglia and astrocytes [12]. These series of events ultimately cause myelin loss and axonal damage. Additionally, many behavioral changes can be observed in the CPZ-fed mice, including changes in motor behavior [13], pain-like symptoms [14], and mental and cognitive deficits [15,16].

Among the different animal models of MS, the CPZ model was found to be more suitable for investigating MS-related cognitive dysfunctions [17]. The pattern of demyelination in the hippocampus and PFC in the CPZ model aligns with MS pattern and physiology [17]. Thus, several studies have shown that rodents fed with CPZ exhibited an impairment in memory. For example, one study revealed that male albino mice fed with CPZ for six weeks showed a decreased alteration in the Y-maze, indicating an impaired spatial memory [18]. Additionally, a study on female Wister rats also displayed an impairment in spatial memory due to CPZ administration for six weeks [18,19]. Moreover, CPZ-fed male mice displayed a reduced preference for the novel object during the recognition phase in the novel object recognition task after three weeks of CPZ administration, suggesting a decrease in cognitive function [20]. In addition to behavioral tasks, histological examination of the hippocampus and the PFC of CPZ-fed animals showed extensive neuronal damage, which was correlated with the neurobehavioral outcomes in female rats [18,19,21].

Several studies suggested the beneficial role of the female sex hormone estrogen (17-β-oestradiol) in enhancing cognition in older women, preventing dementia, and reducing its severity [22,23]. An earlier study reported estragon-induced improvement in cognitive functions including short-term memory impairment, impairments in attention, concentration, and thought processing in a post-menopausal MS woman on hormone replacement therapy [24]. Furthermore, studies showed the therapeutic effects of estrogen on MS in clinical trials [25,26,27] as well as pre-clinical studies on animal models of MS such as experimental autoimmune encephalomyelitis (EAE) [28,29] and the CPZ model [30].

Phytoestrogens, including isoflavones, are dietary compounds derived from plants’ secondary metabolites, they resemble the molecular structure and size of 17-β-oestradiol (E2), the primary female sex hormone. Hence, they are considered a natural alternative of estrogen [31,32]. The role of isoflavones on MS have previously been demonstrated using genistein, a major isoflavone found is soybeans. Genistein showed immunomodulatory effects in EAE [33]. In addition, a recent study found that genistein administration promoted mature oligodendrocytes’ survival in the hippocampus of the CPZ model of MS [34].

Biochanin A (BCA) (4′-methoxy-5 and 7-dihydroxy isoflavone) is an O-methylated isoflavone found in red clover, cabbage, chickpea, and various herbal products [35]. BCA exhibits numerous pharmacological and biological activities such as anti-cancer [36], hepatoprotective [37], anti-microbial [38], antioxidant [39], and anti-inflammatory activities [40]. In addition, several studies explored the effect of BCA on neurodegenerative diseases such as Alzheimer’s disease (AD) [41], Parkinson’s disease (PD) [42] and cerebral ischemia [43]. In an animal model of AD, BCA ameliorated memory and learning decline in scopolamine-challenged male mice [41]. Moreover, BCA diminished behavioral abnormality and prevented microglial activation and neuronal loss in a mouse model of PD induced by lipopolysaccharide [42]. To the best of our knowledge, there is no reported study on the role of BCA on MS, and only few studies investigated its effect on memory and cognitive decline in other neurological conditions [41,44,45].

Several studies suggested the use of male mice in the CPZ model [13,46,47,48,49]. Moreover, an earlier study on Swiss mice showed more severe CPZ-induced demyelination and loss of oligodendrocytes in male Swiss mice compared with female mice [50]. Additionally, several studies used male mice and rats to investigate the effect of BCA in different disease models. [51,52,53,54]. In particular, male mice and rats were used by some studies investigating the neuroprotective effect of BCA [41,42,55]. Thus, this study aimed to investigate the protective effects of BCA on spatial and recognition memory decline in CPZ animal model of MS using Swiss male albino mice.

## 2. Materials and Methods

### 2.1. Animals

Thirty Swiss albino male mice (SWR/J) (18–22 gm) were obtained from the animal house unit, King Fahd Medical Research Center (KFMRC), King Abdulaziz University, Jeddah, Saudi Arabia. Mice were maintained under a 12 h light/dark cycle (light cycle between 7:00 am and 7:00 pm) at an appropriate room temperature (23 ± 2 °C) and humidity (65%). All mice had free access to food and water. Animal experiments were conducted in accordance with the guidelines of the animal unit committee at KFMRC. The study protocol was approved by the biomedical ethics research committee (Approval No. 680-20) at King Abdulaziz University and followed the rules and regulations of the Animal Care and Use Committee at the KFMRC, which complied with the “System of Ethics of Research on Living Creatures” guidelines prepared by King Abdulaziz City for Science and Technology and were approved by Royal Decree No. M/59 dated 24 August 2010.

### 2.2. Drug Preparations

CPZ (C9012-25G) was obtained from Thermo Fisher Scientific (Waltham, MA, USA). To induce acute demyelination, mice were fed for five weeks with 0.2% *w*/*w* of CPZ mixed into ground rodent chow. CPZ mixed chow was prepared daily [56]. BCA (D2016-1G) was purchased from Sigma-Aldrich (St. Louis, MO, USA). The dose of BCA (40 mg/kg) was chosen based on previous studies that tested its effect on Swiss albino mice [41,57]. A stock solution of BCA was prepared daily by mixing BCA powder with DMSO then diluting the mixture with PBS at a ratio of (DMSO: PBS = 1:9) [57]. Each mouse received 0.2 mL of the mixture intraperitoneally (i.p.) daily between 1:00 pm and 3:00 pm. The weights of the mice were measured weekly and doses were adjusted accordingly.

### 2.3. Experimental Design

**Figure 1:**
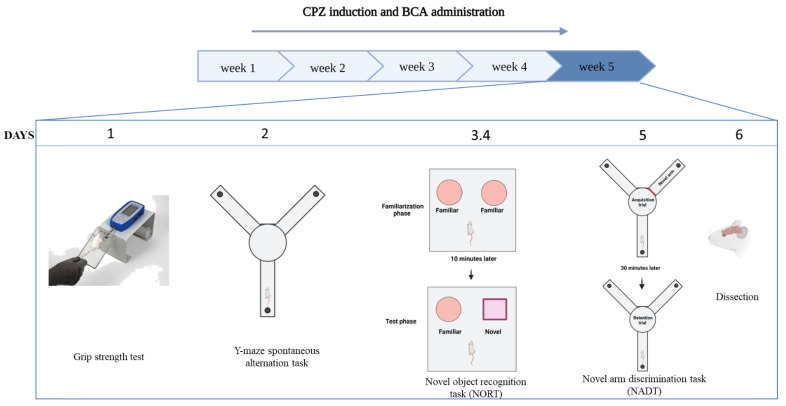
Timescale of the study. The total duration of the study was five weeks. Behavioral tests were performed at the last week of the study. (CPZ: cuprizone; BCA: biochanin A; NORT: novel object recognition test; NADT: novel arm discrimination task).

The total duration of the study was five weeks. Mice were divided randomly into three main groups with 10 mice per group. (1) the control group received 0.2 mL i.p. of 1:9 mixture of DMSO and PBS and normal chow daily for five weeks. (2) the CPZ group received 0.2 mL i.p. of 1:9 mixture of DMSO and PBS- and CPZ-mixed chow daily for five weeks. (3) the CPZ + BCA group received 0.2 mL i.p. of BCA- and CPZ-mixed chow daily for five weeks. Behavioral tests were performed at week 5 (the last week of the study) between 8:00 am and 10:00 am. On test days, mice were habituated to their surroundings for 30 min prior to testing (Figure 1).

### 2.4. Behavioural Tests

#### 2.4.1. Grip Strength Test

To assess neuromuscular function in rodents, the grip strength test was performed. This test determines the maximum peak force developed by a rodent when the experimenter attempts to pull it out of a specially designed grid. The forelimb strength (g force) was measured with a grip strength meter (Columbus Instruments, Columbus, OH, United States). Briefly, mice were held by the tail and were allowed to grip onto the grids by their forelimbs. Once the grip was good, mice were pulled away until the grasp was broken, and the grip-force value was recorded. The test was repeated for three trials per mouse, and the mean values (g) were recorded and normalized to body weight (g/body weight for each mouse) [58].

#### 2.4.2. Y-Maze Spontaneous Alternation Task

Y-maze spontaneous alternation task is commonly used to assess short-term spatial working memory in mice [59]. Animals were placed in a Y-maze apparatus (10 cm width and 40 cm height), composed of three identical arms, and separated equally by 120° angle. Each mouse was placed at one arm end facing the wall of the arm and allowed to explore the maze freely for 8 min. When a mouse visited three arms consecutively an alteration was counted. Arms entries and alternations between the arms were recorded to calculate the percentage of spontaneous alteration following the equation: %alternation = [#alternation/(total arm visit − 2)] [60].

#### 2.4.3. Novel Object Recognition Test (NORT)

To evaluate the effect of CPZ and BCA on short-term recognition memory, NORT was conducted. The protocol was performed as previously described by Labban et al., (2021) [61]. The NORT comprises two phases: the familiarization phase and the test phase. First, mice went through a habituation trial at which each mouse was allowed to explore an empty arena (square test box, 45 × 45 cm) for 3 min. Twenty-four hours later, the familiarization phase was performed. Mice were allowed to explore two identical objects (familiar 1 and familiar 2) placed within the same arena for 3 min per mouse. The test phase was conducted 10 min after the familiarization phase. In this phase, one of the identical objects was replaced with another object (novel object), which is different in shape and color, and animals were allowed to explore the objects for 3 min. The experiment was performed in a noise-free environment, and the objects chosen (familiar and novel) were cleanable and heavy. After each trial, the arena and the objects were cleaned with 10% ethanol to avoid odor cues. The frequency of sniffing (%) was calculated using the equation (novel or familiar object frequency of sniffing/total frequency of sniffing of the two objects × 100) to ensure that all mice had a good chance to explore both objects during the familiarization phase and the test phase. Additionally, the mice’s memory was estimated using the duration of exploration for each object in the test phase by calculating the discrimination index (DI), which is defined as the ability of the mouse to discriminate between novel and familiar objects. DI is calculated according to the following equation: DI = (time spent on the novel object -time spent on the familiar object)/(time spent on the novel object + time spent on the familiar object). All parameters were recorded using an EthoVision tracking system (XT8A system, Noldus Information Technology, Wageningen, The Netherlands) [61].

#### 2.4.4. Novel Arm Discrimination Task (NADT)

To assess spatial recognition memory in mice, NADT was performed using the Y-maze apparatus [62]. The test is composed of two trials: the acquisition trial and the retention trial. In the acquisition trial, one of the three arms was blocked (novel arm) while mice were allowed to explore the other two arms for 5 min. Half an hour later, the blocked arm was opened, and the mice were allowed to explore the maze for 5 min (the retention trial). The floor and the wall of the maze were wiped with 10% alcohol after each mouse to avoid odor cues. Duration spent in the novel arm (%) and percentage of novel arm visits were calculated as an estimate for spatial recognition memory [62].

### 2.5. Histological Examination

To assess the morphological changes in the PFC and the hippocampus, we performed a histological examination using haematoxylin and eosin (H&E) staining. At the end of the experimental period, mice were sacrificed. Brain tissues were carefully removed, rinsed with ice-cold saline, dissected into two hemispheres, and immediately fixed in 10% formalin for 24 h. Tissues were then processed for paraffin embedding, and 5 μm thick sections were prepared and stained with H&E and observed under light microscope [63].

### 2.6. Statistical Analysis

All data are expressed as mean ± standard error of the mean (SEM) and were statistically analyzed using GraphPad Prism 9.1.2. The one-way analysis of variance (ANOVA) followed by a post hoc Tukey’s test was used for comparing differences between the groups in all tests except for frequency of sniffing in which two-way ANOVA followed by post hoc Bonferroni’s test were used. The differences between the groups were considered statistically significant if *p*-value was <0.05.

## 3. Results

### 3.1. BCA Improved the Grip Strength in the CPZ Model

**Figure 2:**
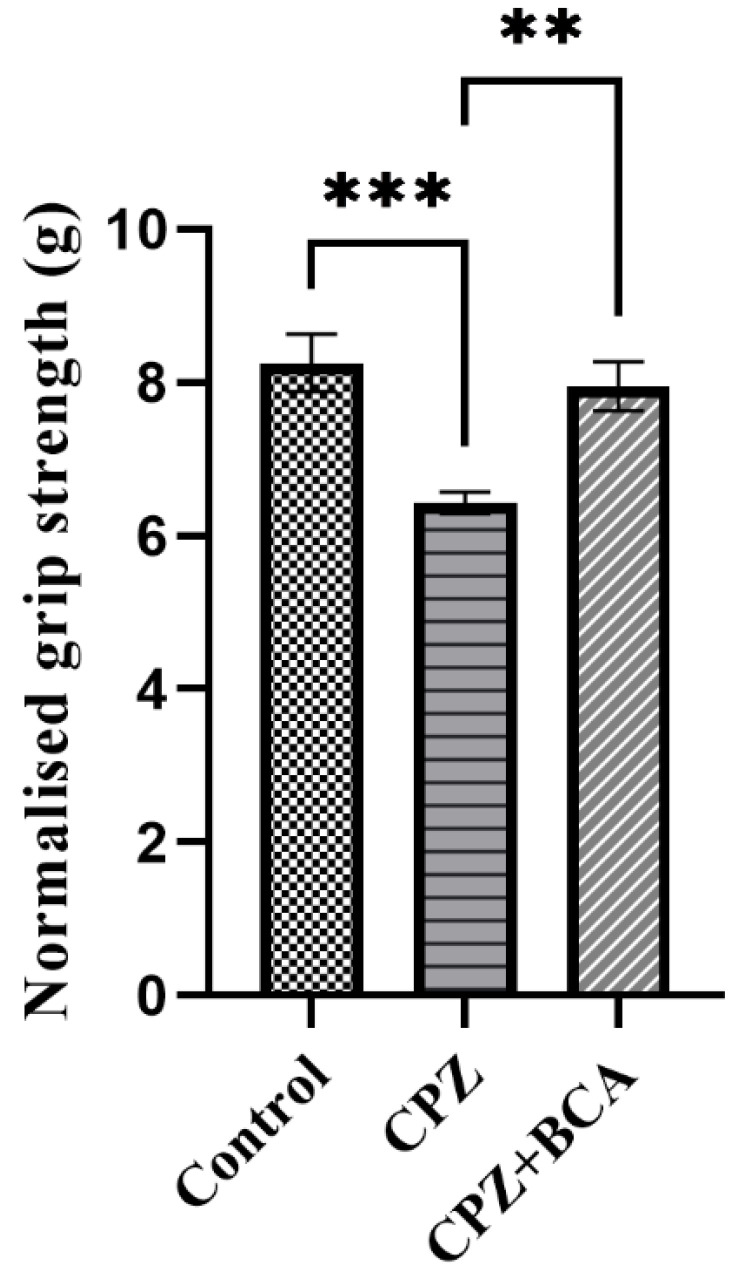
Grip strength test. CPZ significantly reduced the grip strength compared with the control group. BCA significantly increased the grip strength compared with the CPZ group. Data are presented as mean ± SEM. One-way ANOVA followed by Tukey’s test was used. ** *p* < 0.01, *** *p* < 0.001. (CPZ: cuprizone, BCA: biochanin A).

As shown in Figure 2, mice in the CPZ group showed a significant decrease in the grip strength force compared with the control group (*p* = 0.0005). On the other hand, five weeks of BCA administration significantly increased the grip strength compared with the CPZ group (*p* = 0.0034).

### 3.2. BCA Improved the Spontaneous Alteration in the CPZ Model

**Figure 3:**
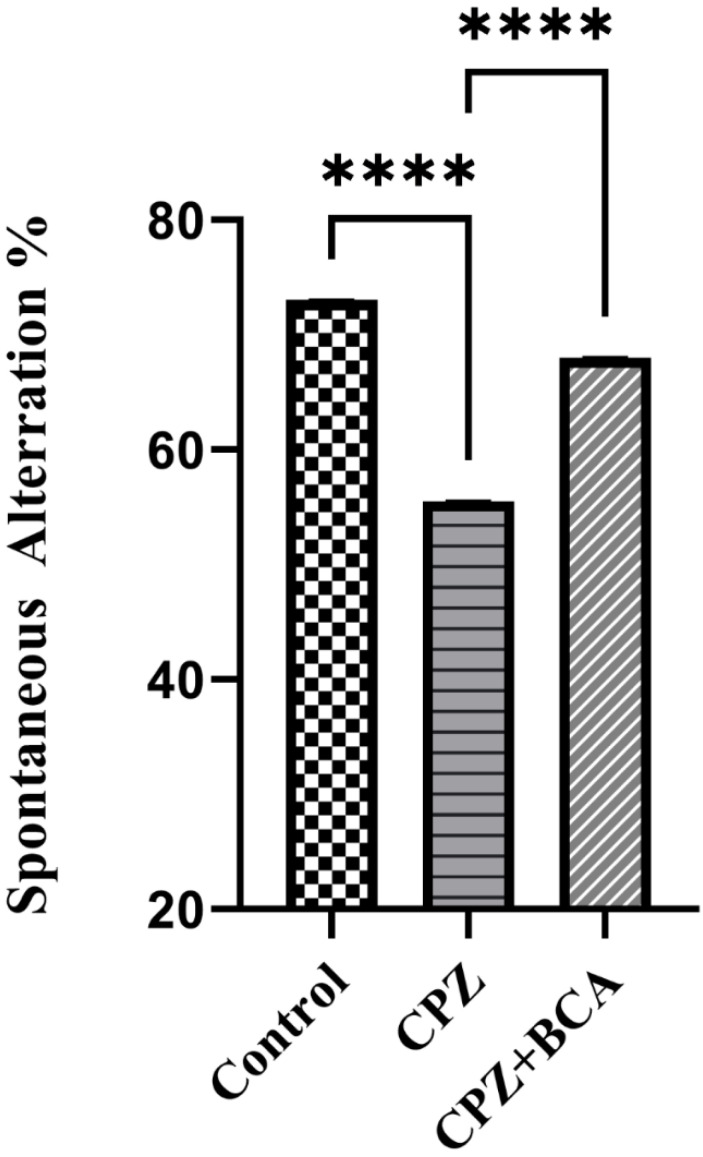
Y-maze spontaneous alteration task. CPZ significantly decreased the alteration % compared with the control group while BCA significantly increased the alteration % in CPZ + BCA group compared with the CPZ group. One-way ANOVA, followed by Tukey’s test was used. Data are present as mean ± SEM. **** *p* < 0.0001. (CPZ: cuprizone, BCA: biochanin A).

CPZ intoxication for five weeks significantly decreased the spontaneous alteration% in the Y-maze compared with the control group (*p* < 0.0001) (Figure 3), whereas BCA administration significantly enhanced the spontaneous alteration % compared with the CPZ group (*p* < 0.0001) (Figure 3).

### 3.3. BCA Improved the DI and the Frequency of Sniffing in CPZ Model

**Figure 4:**
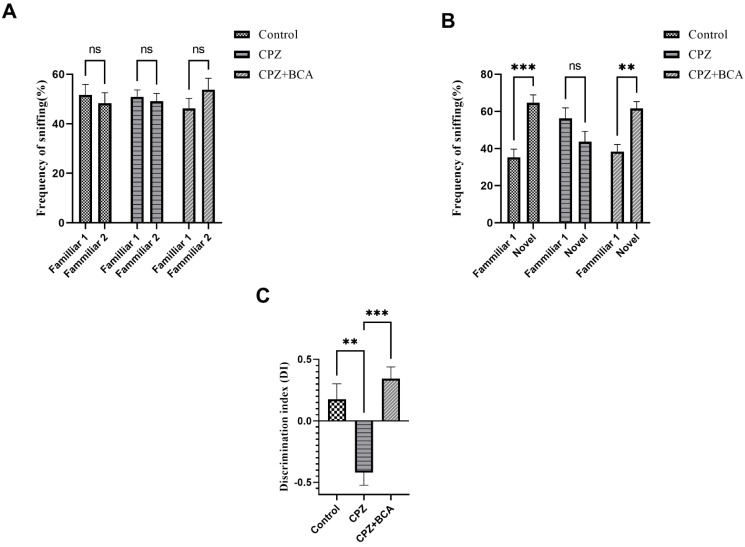
Novel object recognition test (NORT). (**A**) frequency of sniffing (%) during familiarization phase. No significant difference in each group in sniffing each of the familiar objects. (**B**) frequency of sniffing (%) during test phase. Mice in the control group and the BCA group had a significant increase in the frequency of sniffing for the novel object while there was no significant difference in the CPZ group for the frequency of sniffing in the test phase (**C**) DI. CPZ group showed a significant decrease in the DI compared with the control group. BCA significantly improved the DI compared with the CPZ group. Data are presented as mean ± SEM. Two-way ANOVA followed by the Bonferroni multiple comparisons test (**A**,**B**) and one-way ANOVA followed by the Tukey multiple comparisons test (**C**) were used. ns = non-significant, ** *p* < 0.01, *** *p* < 0.001. DI: discrimination index. (CPZ: cuprizone; BCA: biochanin A).

During the familiarization phase of the NORT, there was no significant difference in the frequency of sniffing between the familiar objects (Familiar 1 vs. Familiar 2) among all the groups (control (*p* > 0.999), CPZ (*p* > 0.9999), CPZ + BCA (*p* = 0.5065)) (Figure 4A). During the test phase, the frequency of sniffing of the novel object was significantly higher than the familiar object in the control (*p* = 0001) and BCA groups (*p* = 0.0048), but there was no difference in the CPZ group (*p* = 0.1733) (Figure 4B). In addition, the CPZ group exhibited a significant decrease in the DI compared with the control group (*p* = 0.0019), while the BCA administration group showed a significant increase in the DI compared with the CPZ group (*p* = 0.0001) (Figure 4C).

### 3.4. BCA Improved the Duration in the Novel Arm in the CPZ Model

**Figure 5:**
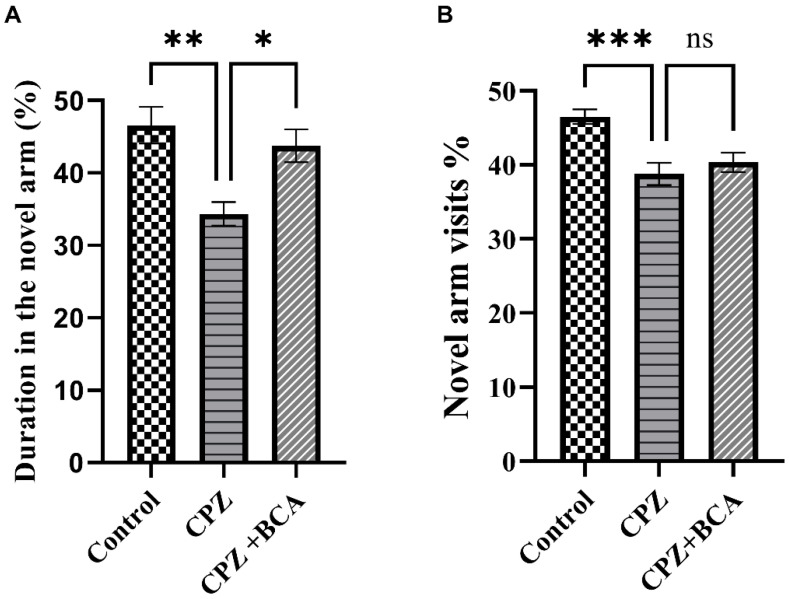
Novel arm discrimination task (NADT). (**A**) Percentage of time in the novel arm: CPZ significantly decreased the duration in the novel arm whereas BCA significantly increased the duration spent in the novel arm compared with the CPZ. (**B**) Percentage of novel arm visits: CPZ significantly decreased the percentage of the novel arm visits compared with the control group, while there was no significant effect of BCA on the percentage of novel arm visits. Data are presented as mean ± SEM. One-way ANOVA followed by Tukey’s test was used. ns = non-significant, * *p* < 0.05, ** *p* < 0.01, *** *p* < 0.001. (CPZ: cuprizone, BCA: biochanin A).

Mice in the CPZ group spent a shorter time in the novel arm and had lesser novel-arm visits compared with the control group, this difference was statistically significant (*p* = 0.0013, *p* = 0.0006, respectively) (Figure 5A,B). On the other hand, BCA administration significantly increased the duration in the novel arm compared with the CPZ group (*p* = 0.0138) (Figure 5A), while it did not show any significant differences in the novel arm visit % (*p* = 0.6583) (Figure 5B).

### 3.5. BCA Mitigated Neuronal Damage in the PFC in the CPZ Model

**Figure 6:**
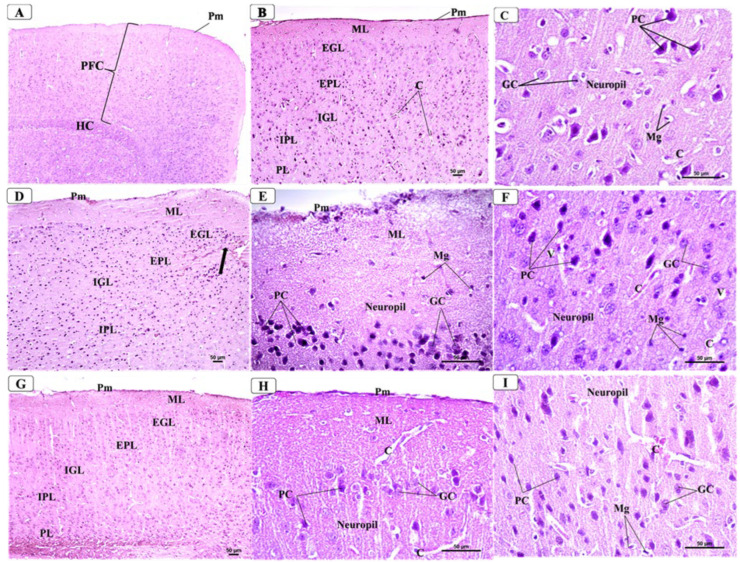
Representative photomicrographs of the PFC: from control group showing: (**A**) General view of PFC covered by thin pia matter (Pm). HC = Hippocampus. ×40 (**B**) Normally arranged layers in the form of molecular layer (ML), external granular layer (EGL), external pyramidal layer (EPL), inner granular layer (IGL), inner pyramidal layer (IPL), and polymorphic layer (PL) from outside to inside. pm = pia matter. ×100 (**C**) Lower layers that contained granular cells with vesicular nuclei (GC) and large pyramidal cells with their vesicular nuclei and basophilic cytoplasm (PC). The neuropil appeared pinkish and showed some microglial cells (Mg) and few capillaries (**C**). (×400). Representative photomicrographs of the PFC from CPZ group showing: (**D**) Disorganized delineation of the cortical layers and thickened pia matter (pm), thickened molecular layer (ML), and hypercellularity (thick arrow) of the external granular layer (EGL) and external pyramidal layer (EPL). ×100. (**E**) Thickened molecular layer (ML) that appeared rarefied and pale due to a smaller number of fibers and cells. Many pyramidal cells (PC) appeared darkly stained and irregular in shape. Some granular cells (GC) showed abnormal shape with darkened cytoplasm × 400 (**F**) Lower layers that contained many pyramidal cells (PC), which were darkly stained and irregular in shape with condensed nuclei and surrounded by vacuolated pale areas. Some granular cells (GC) appeared shrunken and deeply stained. Moreover, many neuroglial cells (Ng) appeared shrunken and deeply stained. ×400. Representative photomicrographs of the PFC from CPZ + BCA group showing: (**G**) Improved organized layers with normally appeared pia matter (pm). ML = molecular layer, EGL = external granular layer, EPL = external pyramidal layer, IGL = internal granular layer, IPL = internal pyramidal layer, and PL = polymorphic layer × 100 (**H**) Nearly normal histological appearance of the upper layers with normally appeared pia matter (pm). ×400 (**I**) Pyramidal cells (PC) that appeared normally or shrunken with dark cytoplasm, blood capillaries (**C**), and neuroglial cells (Ng) were observed. ×400.

Microscopic examination of the H&E-stained sections of the PFC from control mice (Figure 6A–C) showed a normal arrangement formed of six layers from outside to inside; the outer molecular layer was covered with pia mater (few cells and nerve fibers), an external granular layer (granular cells and some small pyramidal cells), an external pyramidal layer (loosely arranged pyramidal cells), an internal granular layer (pyramidal cells and granular cells), an internal pyramidal layer (large pyramidal cells), and a polymorphic layer (fusiform cells and nerve fibers). The pyramidal cells showed vesicular and prominent nucleoli, basophilic cytoplasm, and regular outlines and their processes. The granular cells appeared rounded with open-face prominent nuclei. Moreover, the surrounding background or neuropil appeared pinkish and contained many axons and dendrites of all nerve cells with some blood capillaries and microglial cells. On the other hand, stained sections of CPZ-treated mice (Figure 6D–F) showed distorted delineation of the cortical layers and varied degrees of degenerative changes. The molecular layer was thickened and appeared rarefied and pale due to a smaller number of fibers and cells. Additionally, some neurons in different layers, especially the pyramidal cells, were irregular in shape and condensed with deeply stained shrunken nuclei and the cytoplasm surrounded by pericellular halos. Moreover, many granular cells were distorted in shape, ill-defined, and faintly stained with pericellular halos. Moreover, the neuropil appeared faint and fragmented with the presence of many vacuoles and dilated congested capillaries. Furthermore, microscopic examination of the H&E-stained sections of the PFC from CPZ + BCA-treated mice (Figure 6G–I) showed marked improvement in the morphological appearance as compared with mice that received CPZ only. The pyramidal cells appeared nearly normal with vesicular nuclei while few of them were still condensed with acidophilic cytoplasm. The granular cells were nearly similar as in the control animals, while few cells were more darkly stained, and the neuropil was still vacuolated with the presence of some dilated blood vessels.

### 3.6. BCA Mitigated Neuronal Damage in the Hippocampus in the CPZ Model

#### 3.6.1. Control Group

**Figure 7:**
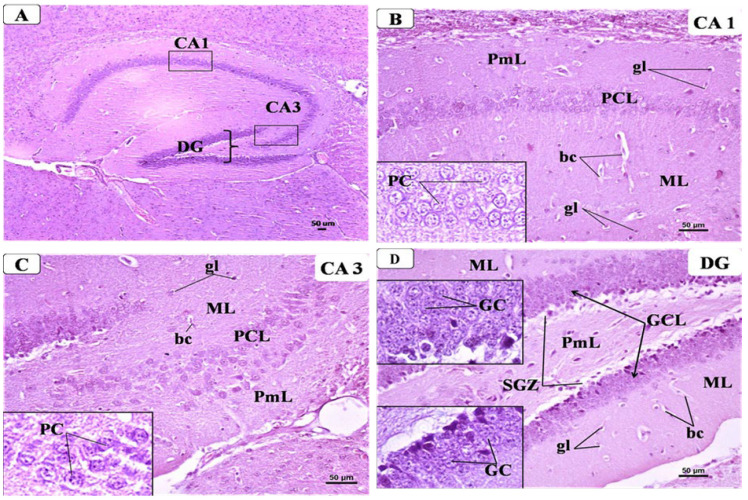
Representative photomicrographs of H&E-stained brain sections showing the hippocampus: from control group: (**A**) C-shaped Cornu Ammonis (CA) with its CA1, CA3, and dentate gyrus (DG). (H&E × 40). (**B**) CA1 was formed of three layers: outer molecular layer (ML), middle pyramidal cell layer (PCL), and inner polymorphic layers (PmL). PCL was formed of well-organized compact layers of small pyramidal cells (PC) containing large vesicular nuclei and pale basophilic cytoplasm (Inset). Both ML and PmL contained some glial cells (gl) with small dark nuclei and perinuclear halos and few blood capillaries (bc) inside the pinkish neuropil matrix. (H&E × 200, inset × 400). (**C**) CA3 was formed of three layers: the molecular layer (ML), pyramidal cell layer (PCL), and polymorphic layers (PmL). PCL was formed of less packed large pyramidal cells with large vesicular nuclei with prominent nucleoli and pale basophilic cytoplasm (inset). Both ML and PmL contained some glial cells (gl) with small dark nuclei and perinuclear halos and few blood capillaries (bc) inside the pinkish neuropil matrix. (H&E × 200, inset × 400). (**D**) DG of control group showing three layers: molecular layer (ML), granular layer (GCL), and polymorphic (PmL) of both upper and lower limbs. Insets displayed GCL, which is the main layer that contained densely packed granular cells (GC) with dark nuclei. Both ML and PmL contained some glial cells (gl) and blood capillaries (bc). A sub-granular zone (SGZ) was seen below GL containing small neurons with oval deeply stained nuclei.

Examination of the hippocampus from the control group displayed its typical shape and structure that formed of Cornu Ammonis and dentate gyrus. Cornu Ammonis was C-shaped and arranged in the following regions: CA1, CA2, CA3, and CA4 (Figure 7A). Each region was composed of three well-defined layers: outer molecular, middle cell pyramidal, and inner polymorphic. The pyramidal layer was the main cellular layer, which was formed of several layers of densely packed small pyramidal cells in CA1 and less densely packed large pyramidal cells in CA3. The pyramidal cells appeared as triangular cells with vesicular nuclei and prominent processes and pale basophilic cytoplasm. Both molecular and polymorphic layers consisted of axons, dendrites, few scattered glial cells, and some blood capillaries on a pink background of neuropil (Figure 7B,C). Meanwhile, the dentate gyrus (DG) in the control group (Figure 7D) appeared as a V-shaped structure enclosing CA4 region by its upper and lower limbs; it was formed of three layers: outer molecular (ML), intermediate granular (GCL), and inner polymorphic (PmL). The granular layer constituted the principal layer, which consisted of closely packed small granular cells with rounded nuclei and a thin rim of cytoplasm. The ML and PmL layers contained few glial cells and blood capillaries. A sub-granular zone (SGZ) was seen below GCL containing small neurons with deeply stained nuclei.

#### 3.6.2. CPZ Group

**Figure 8:**
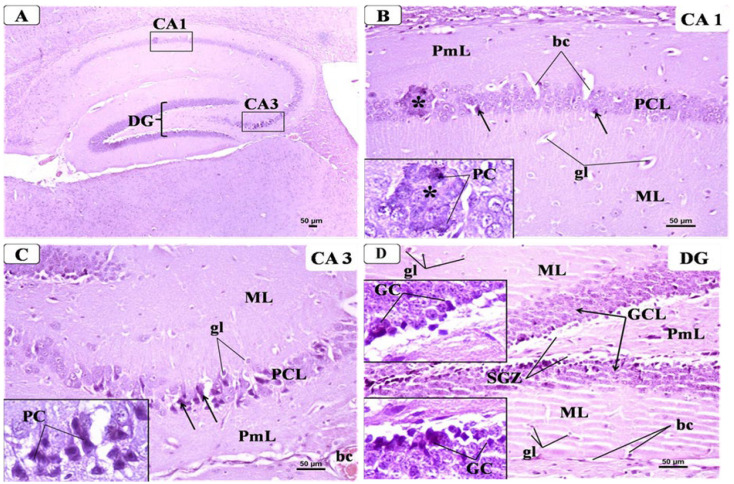
Representative photomicrographs of H&E-stained brain sections showing the hippocampus from the CPZ group: (**A**) C-shaped Cornu Ammonis with its CA1 and CA3 areas and dentate gyrus (DG). (H&E × 40). (**B**) CA1 was formed of three layers: molecular layer (M), pyramidal cell layer (PCL), and polymorphic layer (PmL). There was disarrangement of PCL with some pyramidal cells (PC) appeared shrunken which lost their shape and appeared with dark cytoplasm and small condensed nuclei (arrow). Lower inset showed coalesced and hyalinized PCs. (H&E × 200, Inset × 400.) (**C**) CA3 was formed of three layers; molecular layer (ML), pyramidal cell layer (PCL) and polymorphic layer (PmL). There was disorganization and degenerated pyramidal cells (PC). Lower inset showed PCs with dark cytoplasm and condensed nucleus. (H&E × 200, Inset × 400.) (**D**) DG of the CPZ group showing many degenerated granular cells (GC) with vacuolated cytoplasm and condensed nucleus (insets). Notice the dilated capillaries (bc) and increased glial cells (gl) in ML and PmL layer. Widening of SGZ with few small neurons could be seen.

Examination of the hippocampus from CPZ group revealed the presence of variable changes in different regions of the hippocampal formation. The main changes were seen in the pyramidal cell layer (PCL) of CA1 and CA3 in the form of disorganization and degeneration of many pyramidal cells, which lost their shape and appeared shrunken with dark cytoplasm and condensed nuclei. Moreover, some PCs appeared coalesced and hyalinized. Both ML and PmL layers showed increased glial cells and dilated blood capillaries (Figure 8B,C). Regarding DG in CPZ group (Figure 8D); the main changes occurred in GCL that appeared disorganized with some cell loss. Many degenerated GC appeared shrunken with vacuolated cytoplasm and condensed nucleus. The widening of SGZ with few small neurons could be seen. ML and PmL layers of DG showed increased glial cells with pericellular haloes, dilated blood capillaries, and some vacuolation in their neuropil.

#### 3.6.3. CPZ + BCA Group

**Figure 9:**
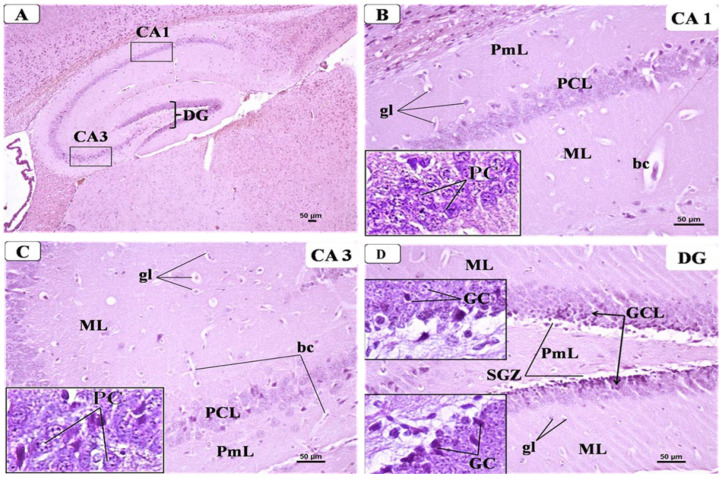
Representative photomicrographs of H&E-stained sections of the hippocampus from CPZ + BCA A-treated group: (**A**) C-shaped Cornu Ammonis with its CA1 and CA3 areas and dentate gyrus (DG). (H&E × 40.) (**B**) CA1 was formed of three layers: ML, PCL, and PmL. There was improvement of the histological picture with the preservation of most of the pyramidal cells (PC) as compared with the CPZ group but few cells showing condensed nuclei (H&E × 200, Inset × 400). (**C**) CA3 was formed of three layers: ML, PCL, and PmL. Many pyramidal cells (PC) looked normal with vesicular nuclei (H&E × 200, Inset × 400). (**D**) DG showing preservation of many granular cells (GC) appeared nearly similar to the control (insets). Both ML and PmL layers contained normally appearing glial cells (gl) and blood capillaries (bc). SGZ appeared nearly normal.

Examination of the hippocampus from CPZ + BCA-treated group (Figure 9A) revealed improved morphology with minimal changes when compared with the CPZ group with relatively normal thickness of the pyramidal layer in CA1 and CA3. Most of the pyramidal cells (PC) were preserved and showing vesicular nuclei with few cells showing dark condensed nuclei. Regarding DG in CPZ + BCA-treated group (Figure 9D), there was apparently normal GC, which were compactly arranged with rounded pale vesicular nuclei. Some appear shrunken (zigzag arrow) and few are dark with pyknotic nuclei in GCL. The ML and PmL appeared such as those of the control group with the normal appearance of glial cells and blood. SGZ contained small neurons.

## 4. Discussion

The present study explored for the first time the possible protective effects of the isoflavone BCA on memory decline in the CPZ animal model of MS. Interestingly, our data shows that BCA significantly ameliorated memory decline by improving behavioral deficits and the morphological changes in the PFC and the hippocampus after five weeks of administration.

About 80% of MS patients exhibit motor deficits that affect muscle movement in the extremities [64]. Thus, to investigate the neurotoxic effect of CPZ, we conducted the grip strength test, which is known to be used to screen neurobehavioral toxicity [65]. Changes in grip strength have been interpreted as evidence of motor neurotoxicity. Our results show that CPZ administration for five weeks caused muscle weakness, as indicated by the decreased strength of forelimbs. This finding is in line with two previous studies that demonstrated the effect of CPZ intoxication on neuromuscular function using an automated grip strength apparatus [65,66]. On the other hand, BCA significantly prevented muscle weakness after five weeks of administration.

To assess the effect of CPZ and BCA on spatial and recognition memory, we performed a number of tests including the Y-maze spontaneous alteration test, the NORT, and the NADT. These groups of behavioral tests depend on animals’ innate desire to explore novelty [59,62,67]. In our study, the CPZ-fed mice exhibited short-term memory impairments as shown by their spatial and recognition performances. In particular, in the Y-maze spontaneous alternation task, mice fed with CPZ showed a significant reduction in the alteration behavior. Meanwhile, in the NORT, CPZ-fed mice failed to discriminate between the novel and familiar objects. Finally, in the NADT, CPZ-intoxicated mice spent less time in the novel arm and had lesser novel arm visits. These findings are in line with previous studies that investigated the effect of CPZ feeding on the Y-maze [10,68,69,70,71], NORT [8,16,20,72,73], and NADT [74]. Taken altogether, our findings indicate a spatial and recognition memory impairment in the CPZ group after five weeks of administration. The effect of CPZ intoxication on spatial performance in the Y-maze was previously correlated with demyelination, microglial activation, and increased levels of the proinflammatory cytokines, as well as tumor necrosis factor alpha (TNF-α) and interleukin 1-beta (IL-1β) in different brain regions, such as the corpus callosum hippocampus and the frontal cortex [10,70]. Additionally, a study by Murakami et al., (2017) showed that recognition memory impairment in mice fed with CPZ was linked to increased inflammatory responses of microglia and astrocytes [16].

On the other hand, BCA administration in our study showed a significant prevention of spatial and recognition memory deterioration in the Y-maze, NORT, and NADT. To the best of our knowledge, this is the first study to assess the protective effect of BCA on the tasks mentioned above. This significant effect of BCA on CPZ-induced neurobehavioral deficit could be attributed to several of its prominent properties, such as its anti-inflammatory activity, antioxidant activity, and anti-apoptotic activity. Zhang and Chen (2015) reported that BCA treatment inhibited the release of inflammatory cytokines such as TNF-α and IL-1β and inflammatory mediators, inducible nitric oxide synthase (iNOS), nitric oxide (NO), and prostaglandin E2 (PGE2) in LPS-induced BV2 microglia cells [40]. Moreover, in a previous study by Wu. et al. (2018), BCA administration enhanced the neurobehavioral function of Sprague–Dawley rats in a modified water maze and provided neuroprotection against early brain injury via suppressing TLR/NF-κB pathway-mediated inflammatory agents [43]. In addition, evidence of the antioxidant and antiapoptotic effects of BCA on memory and learning decline were previously reported. A study by Biradar et al. (2013) showed that BCA administration prevented neurobehavioral deficits by decreasing lipid peroxidation and increasing glutathione (GSH) levels in scopolamine-challenged mice [41]. Moreover, Zhao et al. (2021) stated that BCA administration enhanced learning and memory in ovariectomy-induced cognition by decreasing malondialdehyde (MDA) levels and Bcl-2 expression while increasing GSH, superoxide dismutase (SOD) levels, and Bax and Caspase-3 expression [45]. Thus, we speculate that these BCA activities could have played a major role in preventing behavioral deficits in mice fed with CPZ.

Moreover, we performed histological analysis using H&E staining to observe the morphological changes of the PFC and the hippocampus of our study’s experimental animals. CPZ administration for five weeks compromised neuronal integrity as observed by the degenerative changes in both regions. Pyramidal cells appeared irregular in shape with shrunken and dark nuclei in the PFC and the CA1 and CA3 regions of the hippocampus, and the granular cells appeared degenerated in the dentate gyrus of the hippocampus. Our finding agrees with previous reports that demonstrated the effect of CPZ on the integrity of neuronal cells of the PFC and the hippocampus [18,19,21]. On other hand, histological analysis shows that BCA administration prevented the CPZ-induced degenerative changes in the PFC and the hippocampus of the experimental animals. These findings are supported with previous studies showing the effect of BCA administration on hippocampal neurons in scopolamine-challenged mice and ovariectomy-induced cognition deficits [41,45]. Similarly, the effect of BCA administration on the morphological changes could be explained by BCA previously reported antioxidant and anti-inflammatory activities.

The findings of our study are based on male mice. It would be of great interest to investigate the effect of BCA on CPZ-induced memory decline in female mice or conduct a sex difference study. Previous studies investigating the therapeutic effects of estrogen receptors (Erβ) ligands such as estriol, diarylpropionitrile, and indazole-Cl on EAE model of MS did not show noticeable changes between male and female mice [28,75,76,77]. Since BCA is an Erβ ligand [78], we predict similar findings in male as well as female mice.

## 5. Conclusions and Future Perspective

To our knowledge, our present study is the first work that provides evidence about the possible protective effect of BCA on CPZ-induced memory decline. In summary, our study showed that BCA administration for five weeks improved neurobehavioral deficits in the cognitive tasks, Y-maze spontaneous alteration tasks, NORT, and NADT. Moreover, BCA also mitigated the degenerative changes in the PFC and hippocampus region caused by CPZ feeding.

For future studies, we recommend investigating the effect of different doses of BCA on memory decline in the CPZ model. Moreover, further work is needed to elucidate the exact biochemical role of BCA on CPZ-induced memory deficits through investigating BCA anti-inflammatory, anti-apoptotic, and antioxidant properties. In addition, since there is no study up to date that explored the effect of BCA on demyelination, an immunohistochemistry study is recommended to explore the effect of BCA on myelin status in the MS CPZ model. In addition, it would be quite interesting to investigate the role of BCA and related novel compounds in CPZ-induced animal models based on gender difference.

## Data Availability

Not applicable.
